# Meta-analysis of randomized controlled trials on the efficacy of thoracic epidural anesthesia in preventing atrial fibrillation after coronary artery bypass grafting

**DOI:** 10.1186/1471-2261-12-67

**Published:** 2012-08-19

**Authors:** Wan-Jie Gu, Chun-Yin Wei, De-Qing Huang, Rui-Xing Yin

**Affiliations:** 1 Department of Cardiology, Institute of Cardiovascular Diseases, the First Affiliated Hospital, Guangxi Medical University, Nanning, Guangxi, People’s Republic of China; 2 Department of Anaesthesiology, the First Affiliated Hospital, Guangxi Medical University, Nanning, Guangxi, People’s Republic of China; 3 Department of Encephalopathy, the First Affiliated Hospital, Guangxi Traditional Chinese Medical University, Nanning, Guangxi, People’s Republic of China

**Keywords:** Thoracic epidural anesthesia, Postoperative atrial fibrillation, Meta-analysis

## Abstract

**Background:**

Postoperative atrial fibrillation (POAF) is one of the most common complications in patients undergoing coronary artery bypass grafting (CABG). The goal of this meta-analysis was to evaluate the efficacy of thoracic epidural anesthesia (TEA) in preventing POAF in adult patients undergoing CABG.

**Methods:**

MEDLINE and EMBASE were searched to identify randomized controlled trails in adult patients undergoing CABG who were randomly assigned to receive general anesthesia plus thoracic epidural anesthesia (GA + TEA) or general anesthesia only (GA). Two authors independently extracted data using a standardized Excel file. The primary outcome measure was the incidence of POAF. We used DerSimonian-Laird random-effects models to compute summary risk ratios with 95% confidence intervals.

**Results:**

Five studies involving 540 patients met our inclusion criteria. No significant difference in the incidence of POAF was observed between the two groups (risk ratio, 0.61; 95% confidence interval, 0.33 to 1.12; *P* = 0.11), with significant heterogeneity among the studies (I^2^ = 73%, *P* = 0.005). Sensitivity analyses by primary endpoint, methodological quality and surgical technique yielded similar results.

**Conclusions:**

The limited evidence suggests that TEA shows no beneficial efficacy in preventing POAF in adult patients undergoing CABG. However, the results of this meta-analysis should be interpreted with caution due to significant heterogeneity of the studies included. Thus, the potential infuence of TEA on the incidence of atrial fibrillation following CABG warrants further investigation.

## Background

Postoperative atrial fibrillation (POAF) is one of the most common complications encountered after CABG. The incidence of POAF reported in previous studies varies between 20% and 40%, depending on definitions and methods of detection [1-3]. Although the majority of POAF are benign and self-limiting, it has been shown to increase both the length of hospital stay and total hospital costs significantly [4,5]. Thus, the prevention of POAF is of great importance. In recent decades, many pharmacologic interventions have been used to prevent the development of POAF, for example, β-blockers, sotalol, amiodarone, and magnesium [6]. However, all of them have limited effectiveness and are not free of side effects. The limited efficacies of pharmacologic interventions have stimulated research into alternative prophylactic strategies to prevent POAF.

The etiology of POAF is not widely known and has been related to many risk factors [7]. Increased sympathetic activation appears to be important in the pathogenesis of POAF [8]. As TEA reduces sympathetic activity, it seems to be a promising non-pharmacologic intervention to reduce the incidence of POAF and could be beneficial in patients at increased risk of perioperative arrhythmias [9]. However, the studies regarding TEA in preventing POAF conveyed mixed and inconclusive results [10-19]. We therefore performed a meta-analysis based on relevant randomized controlled trials to evaluate the efficacy of TEA in preventing POAF in adult patients undergoing CABG.

## Methods

### Literature search and selection criteria

MEDLINE and EMBASE (up to March 1, 2012) were searched for randomized controlled trails in adult patients undergoing CABG who were randomly assigned to receive GA + TEA or GA only. Search terms included “thoracic epidural” and “coronary artery”. No language restriction was imposed. The searches were limited to human subjects and randomized controlled trials. In addition, reference lists from the identified articles were manually examined for relevant new articles. This process was performed iteratively until no additional articles could be identified.

We included studies in all languages irrespective of blinding when the following inclusion criteria were met: enrolled adult patients (i.e., 18 yr or older) undergoing CABG; randomly assigned to receive GA + TEA or GA only; reported data on the incidence of POAF; and provided definition and monitoring of POAF. Tials were excluded if they enrolled patients 1) requiring salvage CABG, 2) undergoing CABG and valve surgery at the same time, 3) existing preoperative atrial fibrillation.

### Data extraction and quality assessment

Two authors (WJG and CYW) independently extracted data from all eligible studies using a standardized Excel file. The following data were extracted from each study: first author’s last name, year of publication, number of enrolled patients, surgery type, CABG technique, perioperative management of TEA, the definition and monitoring methods of POAF, and outcome data. Any disagreements were resolved by discussion and consensus.

We assessed the methodological quality of included RCTs using the Jadad scale [20]. The scale consists of three items describing randomization (0–2 points), blinding (0–2 points), and dropouts and withdrawals (0–1 points) in the report of a randomized controlled trial. A score of 1 is given for each of the points described. A further point is obtained where the method of randomization and/or blinding is given and is appropriate; whereas it is inappropriate a point is deducted. Higher scores indicate better reporting. The quality scale ranges from 0 to 5 points. The studies are said to be of low quality if the Jadad score is ≤2 and high quality if the score is ≥3 [21].

### Statistical analysis

We assessed the overall efficacy of TEA in preventing POAF based on the data from included 5 RCTs. The incidence of POAF was treated as dichotomous variables and was expressed as risk ratio (RR) with 95% confidence interval (CI) for each study.

Heterogeneity across studies was tested by using the I^2^ statistic, which is a quantitative measure of inconsistency across studies. Studies with an I^2^ statistic of 25% to 50% are considered to have low heterogeneity, those with an I^2^ statistic of 50% to 75% are considered to have moderate heterogeneity, and those with an I^2^ statistic of >75% are considered to have a high degree of heterogeneity [22]. I^2^ less than 50% has been considered as nonimportant heterogeneity [23]. In case of important heterogeneity, we used the random-effects model. We further conducted sensitivity analyses to assess the robustness of our results and to explore the potential sources of heterogeneity. Sensitivity analyses were done according to the following: (1) primary endpoint; (2) methodological quality; and (3) CABG technique.

Potential publication bias was evaluated by inspecting funnel plots and performing Begg and Egger tests [24,25]. A two-tailed *P*-value of less than 0.05 was judged as statistically significant. All statistical analyses were performed using Review Manager, Version 5.0 (The Nordic Cochrane Centre, The Cochrane Collaboration; Copenhagen, Denmark).

## Results

### Identification of eligible studies

**Figure 1:**
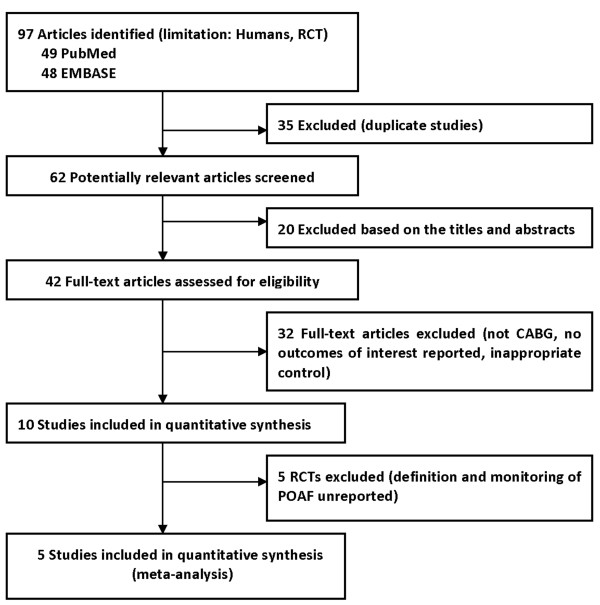
**Selection Process for RCTs Included in the Meta-analysis.** CABG, coronary artery bypass grafting; POAF, postoperative atrial fibrillation; RCT, randomized controlled trials.

A total of 97 studies were identified by the initial database search. Thirty-five studies were excluded because of duplicate studies and 20 studies were excluded based on the titles and abstracts. After detailed assessment, 32 studies were excluded because they did not meet our inclusion criteria. For the remaining 10 RCTs, five of these were also excluded because POAF was not well defined and the monitoring of POAF was not specified clearly [10-14]. Finally, five RCTs that met our inclusion criteria were included in the present meta-analysis [15-19]. The flowchart of studies included in meta-analysis was shown in Figure 1.

### Study characteristics

The summary of RCTs included in the meta-analysis is shown in Table 1. These studies were published between 2001 and 2009. The size of the RCT ranged from 50 to 163 (total 540). Three studies in this meta-analysis enrolled patients undergoing CABG with CPB technique [15,16,18], the remaining two studies included patients undergoing CABG with OPCAB technique [17,19]. The quality of the included studies was assessed by the Jadad score. The median Jadad score of the studies included was 3 (range from 2 to 3).

**Table 1:** Summary of RCTs included in the meta-analysis

**First author/Year of publication**	**No. of subjects**	**Surgery type**	**CABG technology**	**TEA continued postoperatively**	**TEA**	**Perioperative management of TEA**	**Primary endpoint?**	**POAF**		**Study design/Jadad score**
								**TEA + GA**	**GA**	
Jidéus/2001 [15]	121	elective CABG	CPB	Unclear	T2-T5, the day before surgery	Intraoperative: bupivacaine 5 mg/mL, with an infusion rate of 4 to 8 mL/h. Postoperative: continuous infusion of bupivacaine (2 mg/mL) and sufentanil (1 mg/mL) epidurally (3 to 7 mL/h)	Yes	13/41	29/80	RCT/2
Nygard/2004 [16]	163	elective CABG	CPB	4 days	T1-T3, the day before surgery	Intraoperative and postoperative : bolus doses of 4 mL of bupivacaine, 5 mg/mL, given hourly	Yes	28/79	25/84	RCT/3
Bakhtiary/2007 [17]	132	elective CABG	OPCAB	3 days	T1-T3, the day before surgery	Preoperative and postoperative: continuous infusion with ropivacaine 0.16 % and sufentanil 1 g/mL at an hourly rate of 2 to 5 mL was started after a bolus dose of 6 mL	Yes	2/66	18/66	RCT/2
Tenenbein/2008 [18]	50	elective or semi-elective CABG	CPB	2 days	T2-T5, at least four hours prior to systemic heparinization	Intraoperative: 5-mL bolus of 0.75% ropivacaine and 200 μg of hydromorphone in the epidural catheter, followed by infusion of 0.75 % ropivacaine at 5 mL/h. Postoperative: continuous infusion lasting 48 h and consisting of 0.2 % ropivacaine, with 15 μg/mL of hydromorphone titrated.	No	6/25	9/25	RCT/3
Caputo/2009 [19]	74	elective CABG	OPCAB	3 days	T2-T4, before induction of anesthesia	Intraoperative and postoperative: continuous infusion of 0.125 % bupivacaine and 0.0003 % clonidine (150 g in 500 mL) at an initial rate of 10 mL/h	No	7/36	18/38	RCT/3

CABG, coronary artery bypass surgery; OPCAB, off-pump coronary artery bypass grafting; CPB, cardiopulmonary bypass; TEA, thoracic epidural anesthesia; GA, general anesthesia; POAF, postoperative atrial fibrillation; RCT, randomized controlled trial.

### TEA and POAF

**Figure 2:**
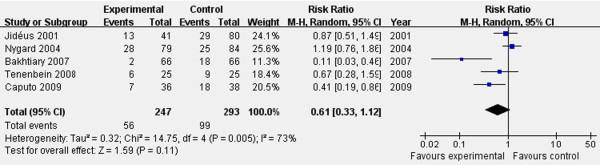
**Forest plot for the incidence of POAF. There were no significant effects in preventing POAF as determined by the random-effects model.** RR, relative risk; CI, confidence interval.

The definition and monitoring of POAF in the various studies are summarized in Table 2. Overall, 540 patients were included in this analysis (247 in the TEA + GA group and 293 in the GA only group). Meta-analysis of 5 studies using a random-effects model suggested that TEA had no significant effect on the prevention of POAF (RR 0.61, 95% CI 0.33 to 1.12; *P* = 0.11; Figure 2). There was significant heterogeneity among the studies (I^2^ = 73%, *P* = 0.005; Figure 2).

**Table 2:** Definition and monitoring of POAF used in the included trials of the meta-analysis

**First author/Year of publication**	**Definition of POAF**	**Monitoring of POAF**
Jideus/2001 [15]	The absence of consistent P waves before each QRS complex and with an irregular ventricular rate, lasting 30 seconds or longer	Twenty-four hour Holter ECG performed on the first 4 consecutive days, or until clinically documented sustained atrial fibrillation
Nygard/2004 [16]	New-onset atrial fibrillation (irrespective of treatment), an irregular narrow complex rhythm with absence of P waves	Twenty-four hour Holter ECG performed on the first 5 consecutive days
Bakhtiary/2007 [17]	An episode of atrial fibrillation or flutter lasting for more than 30 seconds	Continuous ECG performed for 48 hours, then, twice daily 12-lead ECG performed until hospital discharge
Tenenbein/2008 [18]	A need for pharmacologic treatment	Holter ECG performed on the first three postoperative days
Caputo/2009 [19]	New onset of atrial fibrillation not present preoperatively	Continuous ECG performed in the intensive care unit, high-dependency unit, and ward, and daily 12-lead ECG analysis

POAF, postoperative atrial fibrillation; ECG, electrocardiogram.

### Sensitivity analyses

Subsequently, we performed sensitivity analyses to explore the source of this heterogeneity and to examine the influence of various exclusion criteria on the combined estimates. Exclusion of 2 studies in which POAF did not served as the primary endpoint yielded similar results (RR 0.64, 95% CI 0.26 to 1.58; *P* = 0.33), with substantial evidence of heterogeneity (I^2^ = 82%, *P* = 0.004) [18,19]. After exclusion of 2 studies with low-quality studies (Jadad score ≤ 2), the results were still maintained (RR 0.73, 95% CI 0.37 to 1.44; *P* = 0.36), yet heterogeneity was still present (I^2^ = 68%, *P* = 0.04) [15,17]. Exclusion of 2 studies that conducted in patients undergoing CABG with OPCAB technique did not change the pooled results (RR 0.98, 95% CI 0.72 to 1.35; *P* = 0.92), but no evidence of heterogeneity was observed among the remaining studies (I^2^ = 0%, *P* = 0.44) [17,19].

### Publication bias

Publication bias was not assessed because of the limited number (below 10) of studies included in each analysis.

## Discussion

In the present meta-analysis, we have reviewed the literature regarding the efficacy of TEA in preventing POAF in adult patients undergoing CABG. The pooled results from meta-analysis of five RCTs using a random-effects model suggest that TEA shows no beneficial efficacy in preventing POAF in adult patients undergoing CABG. Also, substantial heterogeneity across the studies was observed.

The main finding of our meta-analysis seems to contradict a previous review on the topic, which assessed the effects of TEA on the clinical outcomes in patients undergoing cardiac surgery. In detail, in a meta-analysis by Svircevic et al. [26], it was noted that “the use of TEA in patients undergoing cardiac surgery reduces the risk of postoperative supraventricular arrhythmias.” In fact, the contribution may not be conclusive. As their authors clearly emphasized, the included studies were heterogeneous and the study by Scott et al. [27] in 420 patients mainly contributed to this result. In this study, all patients discontinued β-blockers therapy during the study period. Moreover, the patients in TEA + GA group received clonidine, while the patients in GA group were not given this cardioprotective drug [28]. The above factors may result in the beneficial efficacy of TEA on supraventricular arrhythmias.

For the current meta-analysis, we evaluated the efficacy of TEA in preventing POAF in adult patients undergoing CABG. In an attempt to produce robust results, we pre-stated rigorous inclusion criteria and included only RCTs that provided definition and monitoring of POAF. We found that there was no significant difference in the incidence of POAF between the two groups, but significant heterogeneity was observed among these studies. Our sensitivity analyses suggest that 2 studies conducted in patients undergoing CABG with OPCAB technique probably contributed to the heterogeneity [17,19]. In addition, sensitivity analyses based other various exclusion criteria did not materially alter the pooled results, which added robustness to our main finding.

Our study provides additional interesting clues that may be useful for future research on the topic. Two RCTs included in our meta-analysis were conducted in patients undergoing CABG with OPCAB technique instead of CPB [17,19]. These two studies consistently suggest that TEA + GA significantly reduced the incidence of POAF. Thus, a new question arise, does TEA really reduce the incidence of POAF in patients undergoing CABG with OPCAB technique? However, a recent meta-analysis indicated that OPCAB technique was associated with lower incidence of POAF when compared with CPB technique in the population undergoing CABG [29]. Besides, in one study [17], the patients in the TEA group received ropivacaine which has a substantial anti-inflammatory effect [30]. In the other study [19], the epidural infusion protocol used was similar to the one used by Scott and colleagues [27] and clonidine was administered only to the patients in TEA group. These may make the isolated effects of TEA on the incidence of POAF become less clear and raise additional concerns on the interpretation of the positive results.

One could expect that use of TEA may cause potential complications in patients undergoing CABG. TEA may give rise to the following possible hazardous complications, such as the appearance of epidural hematoma. Systematic anticoagulation needed during cardiopulmonary bypass could increase the incidence of epidural hematoma related to the use of an epidural catheter [31]. In addition, the intense sympathycolysis may lead to systemic hypotension, which can be difficult to correct. In the included studies, no cases of epidural hematoma were reported because this devastating complication is too rare to evaluate in randomized studies. Additional studies or data regarding the potential complications related to TEA are warranted.

There are several potential limitations that should be taken into account. First, substantial heterogeneity among studies was observed. Nevertheless, we were able to detect the major source of heterogeneity through the sensitivity analyses. Second, our analysis was based on only five RCTs and all of them were carried out in only western countries and just enrolled older age patients undergoing elective surgery. Thus, the results of the RCTs need to be reproduced in other populations. Morever, we only included the trial providing definition and monitoring of POAF. Because the end-point of our study was less POAF, the monitoring needs to specificied clearly for the various studies. Since identification of POAF is critical to the conclusion, the monitoring has to be standardized or at least specified. Otherwise the conclusions are hard to justify. Exclusion of the studies lacking a clear definition of POAF may have potential influence on the final analysis. Finally, these studies lack homogeneity in both the method of postoperative monitoring and in their definition of POAF. This may lead to potential underestimation and/or overestimation of the true incidence of POAF.

## Conclusion

In summary, the limited evidence suggests that TEA shows no beneficial efficacy in preventing POAF in adult patients undergoing CABG. However, the results of this meta-analysis should be interpreted with caution due to significant heterogeneity of the studies included. Thus, the potential infuence of TEA on the incidence of atrial fibrillation following CABG warrants further investigation.

## Abbreviations

CABG: Coronary Artery Bypass Grafting; CI: Confidence Interval; CPB: Cardiopulmonary Bypass; ECG: Electrocardiogram; GA: General Anesthesia; OPCAB: Off-Pump Coronary Artery Bypass; POAF: Postoperative Atrial Fibrillation; RCT: Randomized Controlled Trials; RR: Risk Ratio; TEA: Thoracic Epidural Anesthesia.

## Competing interests

The authors declare that they have no competing interests.

## Authors’ contributions

WJG conceived the study, participated in the design, collected the data, and drafted the manuscript. CYW collected the data, and performed statistical analyses. DQH helped to collect the data. RXY conceived the study, participated in the design, and helped to draft the manuscript. All authors read and approved the final manuscript.

## Pre-publication history

The pre-publication history for this paper can be accessed here:

http://www.biomedcentral.com/1471-2261/12/67/prepub

## References

[B1] Mathew JP Parks R Savino JS Friedman AS Koch C Mangano DT Browner WS Atrial fibrillation following coronary artery bypass graft surgery: predictors, outcomes, and resource utilization. MultiCenter Study of Perioperative Ischemia Research Group JAMA 1996 276 300 306 10.1001/jama.1996.03540040044031 8656542

[B2] Frost L Mølgaard H Christiansen EH Jacobsen CJ Pilegaard H Thomsen PE Atrial ectopic activity and atrial fibrillation/flutter after coronary artery bypass surgery. A case-base study controlling for confounding from age, beta-blocker treatment, and time distance from operation Int J Cardiol 1995 50 153 162 10.1016/0167-5273(95)93684-K 7591326

[B3] Zaman AG Archbold RA Helft G Paul EA Curzen NP Mills PG Atrial fibrillation after coronary artery bypass surgery: a model for preoperative risk stratification Circulation 2000 101 1403 1408 10.1161/01.CIR.101.12.1403 10736284

[B4] Mauldin PD Weintraub WS Becker ER Predicting hospital costs for first-time coronary artery bypass grafting from preoperative and postoperative variables Am J Cardiol 1994 74 772 775 10.1016/0002-9149(94)90432-4 7942547

[B5] Aranki SF Shaw DP Adams DH Rizzo RJ Couper GS VanderVliet M Collins JJ Jr Cohn LH Burstin HR Predictors of atrial fibrillation after coronary artery surgery. Current trends and impact on hospital resources Circulation 1996 94 390 397 10.1161/01.CIR.94.3.390 8759081

[B6] Burgess DC Kilborn MJ Keech AC Interventions for prevention of post-operative atrial fibrillation and its complications after cardiac surgery: a meta-analysis Eur Heart J 2006 27 2846 2857 10.1093/eurheartj/ehl272 17015402

[B7] Echahidi N Pibarot P O'Hara G Mathieu P Mechanisms, prevention, and treatment of atrial fibrillation after cardiac surgery J Am Coll Cardiol 2008 51 793 801 10.1016/j.jacc.2007.10.043 18294562

[B8] Kalman JM Munawar M Howes LG Louis WJ Buxton BF Gutteridge G Tonkin AM Atrial fibrillation after coronary artery bypass grafting is associated with sympathetic activation Ann Thorac Surg 1995 60 1709 1715 10.1016/0003-4975(95)00718-0 8787468

[B9] Scott NB Turfrey DJ Ray DA Nzewi O Sutcliffe NP Lal AB Norrie J Nagels WJ Ramayya GP A prospective randomized study of the potential benefits of thoracic epidural anesthesia and analgesia in patients undergoing coronary artery bypass grafting Anesth Analg 2001 93 528 535 10.1097/00000539-200109000-00003 11524314

[B10] Priestley MC Cope L Halliwell R Gibson P Chard RB Skinner M Klineberg PL Thoracic epidural anesthesia for cardiac surgery: the effects on tracheal intubation time and length of hospital stay Anesth Analg 2002 94 275 282 11812684 10.1097/00000539-200202000-00009

[B11] Fillinger MP Yeager MP Dodds TM Fillinger MF Whalen PK Glass DD Epidural anesthesia and analgesia: effects on recovery from cardiac surgery J Cardiothorac Vasc Anesth 2002 16 15 20 10.1053/jcan.2002.29639 11854872

[B12] de Vries AJ Mariani MA van der Maaten JM Loef BG Lip H To ventilate or not after minimally invasive direct coronary artery bypass surgery: the role of epidural anesthesia J Cardiothorac Vasc Anesth 2002 16 21 26 10.1053/jcan.2002.29645 11854873

[B13] Royse C Royse A Soeding P Blake D Pang J Prospective randomized trial of high thoracic epidural analgesia for coronary artery bypass surgery Ann Thorac Surg 2003 75 93 100 10.1016/S0003-4975(02)04074-2 12537199

[B14] Barrington MJ Kluger R Watson R Scott DA Harris KJ Epidural anesthesia for coronary artery bypass surgery compared with general anesthesia alone does not reduce biochemical markers of myocardial damage Anesth Analg 2005 100 921 928 10.1213/01.ANE.0000146437.88485.47 15781499

[B15] Jidéus L Joachimsson PO Stridsberg M Ericson M Tydén H Nilsson L Blomström P Blomström-Lundqvist C Thoracic epidural anesthesia does not influence the occurrence of postoperative sustained atrial fibrillation Ann Thorac Surg 2001 72 65 71 10.1016/S0003-4975(01)02631-5 11465233

[B16] Nygård E Sørensen LH Hviid LB Pedersen FM Ravn J Thomassen L Svendsen JH Eliasen K Krogsgaard K Aldershvile J Effects of amiodarone and thoracic epidural analgesia on atrial fibrillation after coronary artery bypass grafting J Cardiothorac Vasc Anesth 2004 18 709 714 10.1053/j.jvca.2004.08.006 15650978

[B17] Bakhtiary F Therapidis P Dzemali O Ak K Ackermann H Meininger D Kessler P Kleine P Moritz A Aybek T Dogan S Impact of high thoracic epidural anesthesia on incidence of perioperative atrial fibrillation in off-pump coronary bypass grafting: a prospective randomized study J Thorac Cardiovasc Surg 2007 134 460 464 10.1016/j.jtcvs.2007.03.043 17662790

[B18] Tenenbein PK Debrouwere R Maguire D Duke PC Muirhead B Enns J Meyers M Wolfe K Kowalski SE Thoracic epidural analgesia improves pulmonary function in patients undergoing cardiac surgery Can J Anaesth 2008 55 344 350 10.1007/BF03021489 18566197

[B19] Caputo M Alwair H Rogers CA Ginty M Monk C Tomkins S Mokhtari A Angelini GD Myocardial, inflammatory, and stress responses in off-pump coronary artery bypass graft surgery with thoracic epidural anesthesia Ann Thorac Surg 2009 87 1119 1126 10.1016/j.athoracsur.2008.12.047 19324137

[B20] Jadad AR Moore RA Carroll D Jenkinson C Reynolds DJ Gavaghan DJ McQuay HJ Assessing the quality of reports of randomized clinical trials: is blinding necessary? Control Clin Trials 1996 17 1 12 10.1016/0197-2456(95)00134-4 8721797

[B21] Kjaergard LL Villumsen J Gluud C Reported methodologic quality and discrepancies between large and small randomized trials in meta-analyses Ann Intern Med 2001 135 982 989 11730399 10.7326/0003-4819-135-11-200112040-00010

[B22] Higgins JP Thompson SG Deeks JJ Altman DG Measuring inconsistency in meta-analyses BMJ 2003 327 557 560 10.1136/bmj.327.7414.557 12958120 PMC192859

[B23] Armitage P Berry G Matthews JNS Analysing Means and Proportions. Statistical Methods in Medical Research 2002 Blackwell Science, Oxford 83 146

[B24] Begg CB Mazumdar M Operating characteristics of a rank correlation test for publication bias Biometrics 1994 50 1088 1101 10.2307/2533446 7786990

[B25] Egger M Davey Smith G Schneider M Minder C Bias in meta-analysis detected by a simple, graphical test BMJ 1997 315 629 634 10.1136/bmj.315.7109.629 9310563 PMC2127453

[B26] Svircevic V van Dijk D Nierich AP Passier MP Kalkman CJ van der Heijden GJ Bax L Meta-analysis of thoracic epidural anesthesia versus general anesthesia for cardiac surgery Anesthesiology 2011 114 271 282 10.1097/ALN.0b013e318201d300 21239975

[B27] Scott NB Turfrey DJ Ray DA Nzewi O Sutcliffe NP Lal AB Norrie J Nagels WJ Ramayya GP A prospective randomized study of the potential benefits of thoracic epidural anesthesia and analgesia in patients undergoing coronary artery bypass grafting Anesth Analg 2001 93 528 535 10.1097/00000539-200109000-00003 11524314

[B28] Wijeysundera DN Bender JS Beattie WS Alpha-2 adrenergic agonists for the prevention of cardiac complications among patients undergoing surgery Cochrane Database Syst Rev 2009 7 CD004126 19821319 10.1002/14651858.CD004126.pub2

[B29] Panesar SS Athanasiou T Nair S Rao C Jones C Nicolaou M Darzi A Early outcomes in the elderly: a meta-analysis of 4921 patients undergoing coronary artery bypass grafting–comparison between off-pump and on-pump techniques Heart 2006 92 1808 1816 10.1136/hrt.2006.088450 16775087 PMC1861313

[B30] Blumenthal S Borgeat A Pasch T Reyes L Booy C Lambert M Schimmer RC Beck-Schimmer B Ropivacaine decreases inflammation in experimental endotoxin-induced lung injury Anesthesiology 2006 104 961 969 10.1097/00000542-200605000-00012 16645448

[B31] Ho AM Chung DC Joynt GM Neuraxial blockade and hematoma in cardiac surgery: estimating the risk of a rare adverse event that has not (yet) occurred Chest 2000 117 551 555 10.1378/chest.117.2.551 10669702

